# Design, formulation and evaluation of novel dissolving microarray patches containing a long-acting rilpivirine nanosuspension

**DOI:** 10.1016/j.jconrel.2018.11.002

**Published:** 2018-12-28

**Authors:** Maelíosa T.C. Mc Crudden, Eneko Larrañeta, Annie Clark, Courtney Jarrahian, Annie Rein-Weston, Sophie Lachau-Durand, Nico Niemeijer, Peter Williams, Clement Haeck, Helen O. McCarthy, Darin Zehrung, Ryan F. Donnelly

**Affiliations:** a School of Pharmacy, Queen's University Belfast, 97 Lisburn Road, Belfast BT9 7BL, UK; b PATH, 2201 Westlake Avenue, Seattle, Washington 98121, USA; c Janssen Pharmaceutica, Turnhoutseweg 30, 2340 Beerse, Belgium

**Keywords:** HIV, Microarray patch, Rilpivirine, Antiretroviral, HIV, Human immunodeficiency virus, AIDS, acquired immune deficiency syndrome, ARV, antiretroviral, PrEP, pre-exposure prophylaxis, NNRTI, non-nucleoside reverse transcriptase inhibitor, MAP, microarray patch, RPV LA, rilpivirine long-acting nanosuspension, PVP, poly(vinylpyrrolidone), PVA, poly(vinyl alcohol)

## Abstract

One means of combating the spread of human immunodeficiency virus (HIV) is through the delivery of long-acting, antiretroviral (ARV) drugs for prevention and treatment. The development of a discreet, self-administered and self-disabling delivery vehicle to deliver such ARV drugs could obviate compliance issues with daily oral regimens. Alternatives in development, such as long-acting intramuscular (IM) injections, require regular access to health care facilities and disposal facilities for sharps. Consequently, this proof of concept study was developed to evaluate the use of dissolving microarray patches (MAPs) containing a long-acting (LA) nanosuspension of the candidate ARV drug, rilpivirine (RPV). MAPs were mechanically strong and penetrated skin *in vitro*, delivering RPV intradermally. In *in vivo* studies, the mean plasma concentration of RPV in rats (431 ng/ml at the Day 7 time point) was approximately ten-fold greater than the trough concentration observed after a single-dose in previous clinical studies. These results are the first to indicate, by the determination of relative exposures between IM and MAP administration, that larger multi-array dissolving MAPs could potentially be used to effectively deliver human doses of RPV LA. Importantly, RPV was also detected in the lymph nodes, indicating the potential to deliver this ARV agent into one of the primary sites of HIV replication over extended durations. These MAPs could potentially improve patient acceptability and adherence to HIV prevention and treatment regimens and combat instances of needle-stick injury and the transmission of blood-borne diseases, which would have far-reaching benefits, particularly to those in the developing world.

## Graphical abstract

**Figure:**
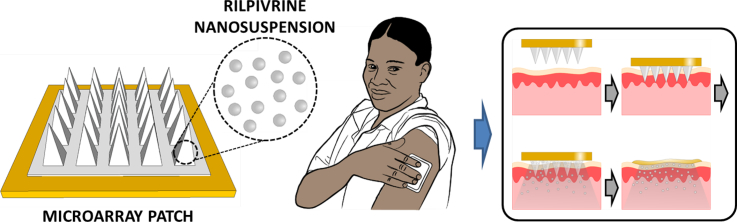

## 1 Introduction

Antiretroviral (ARV) drugs have significantly decreased human immunodeficiency virus (HIV) and acquired immune deficiency syndrome (AIDS) related mortality and morbidity [1]. In high-income countries, the life expectancy of people living with HIV/AIDS now approaches that of the HIV-negative population, but this is not the case in sub-Saharan Africa, where AIDS is still the leading cause of mortality among adults 15 to 59 years of age. Women are increasingly bearing a disproportionate burden of the AIDS epidemic [2,3]. One means of addressing this burden is through efforts to develop long-acting ARV drug delivery systems for HIV prevention that could be appropriate for use by those at greatest risk of infection. Current guidelines for HIV treatment recommend daily usage of oral ARV drugs but many patients experience treatment fatigue, thus affecting regimen adherence. The development of long-acting (LA) ARV drugs and delivery systems promises to address and potentially combat these issues surrounding treatment fatigue and poor adherence [4].

**Fig. 1:**
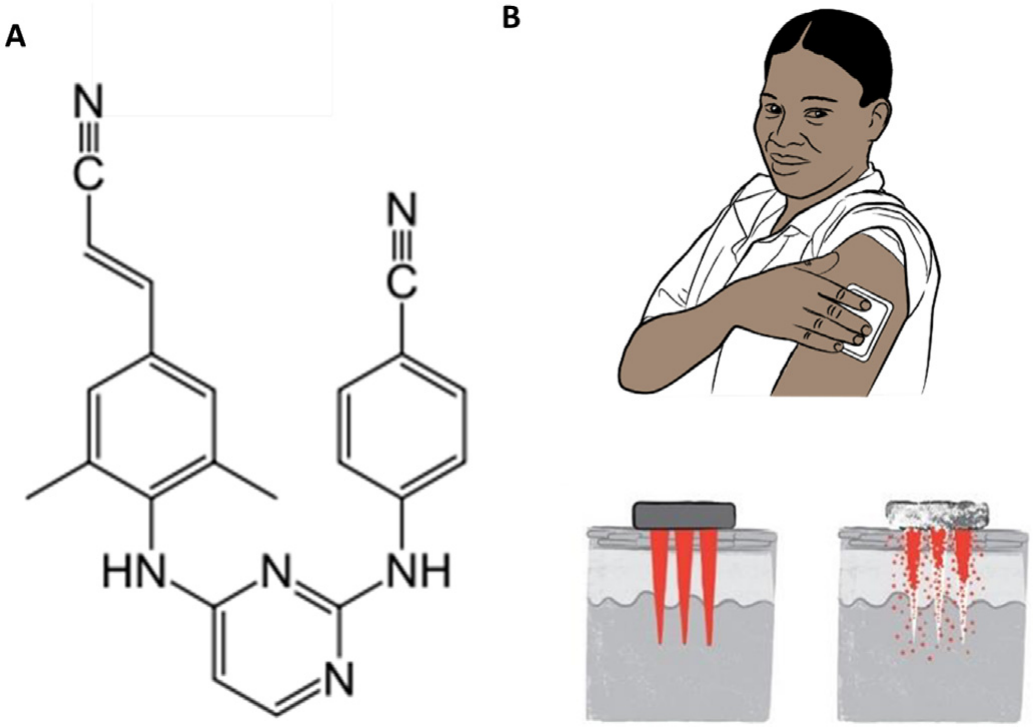
The chemical structure of RPV (A). Schematic representation of the mode of RPV LA MAP application and subsequent in-skin dissolution of the RPV LA MAP (B).

Rilpivirine (RPV) is a next-generation non-nucleoside reverse transcriptase inhibitor (NNRTI) that exhibits potent activity against wild-type HIV and some HIV mutants resistant to first-generation NNRTIs and has been approved for treatment since 2011 [[5], [6], [7]]. The standard adult oral dosage of RPV is a 25 mg tablet, daily [5,8,9]. The physiochemical (Fig. 1A) and pharmacological properties of RPV have enabled development of a long-acting RPV nanosuspension (RPV LA) by Janssen Pharmaceutical, aimed at overcoming onerous daily oral dosing regimens [10]. This innovative intramuscular (IM) RPV formulation requires infrequent injections, at four or eight week intervals, is well tolerated in animals and humans and elicits sustained drug concentrations in systemic circulation [8]. The administration of RPV LA *via* the IM route, however, requires administration by a trained health care worker, which may limit access. Pain at the injection site has been reported in clinical studies, which could impact acceptability of a future product intended for use at either four or eight week intervals [7]. Misuse and inappropriate disposal of needles are also problems of particular significance in the developing world, potentially leading to the transmission of blood-borne diseases. Recent studies carried out over 6–12 month periods assessing the frequency of needle-stick injuries in clinical settings have shown the prevalence of such injuries in health care workers to be high over the specific reporting periods of the studies: 51% in Benin City, Nigeria [11]; 52.7% in Asaba, Nigeria [12], 83.8% in Minna, Nigeria [13] and 86.1% in New Delhi, India [14]. Not all needle-stick injuries are reported, with estimates suggesting that between 40%–75% of such injuries are never reported [14].

The work presented here provides proof of principle for the use of an alternative, needle-free delivery system for LA nanosuspensions in the treatment of HIV, specifically focusing on determining the potential to use novel dissolving microarray patch (MAP) technologies [15] to facilitate intradermal delivery of RPV LA. MAPs are micron-scale devices, which can painlessly pierce the outermost layer of the skin, the *stratum corneum*, to facilitate intradermal delivery of drugs and vaccines [16,17]. MAPs have never before been used to facilitate intradermal delivery of LA nanosuspensions but their potential has previously been lauded in the literature [18] and is represented schematically in Fig. 1B.

Infrequent application of MAPs could potentially obviate compliance issues with conventional delivery strategies, such as daily oral dosing regimens or IM injections, which require delivery by health care providers. This approach indicates that RPV, delivered *via* MAP, could be deposited as a depot into the skin and as the nanoparticles dissolve in interstitial fluid, the drug would slowly be released and absorbed into the systemic circulation. If sufficient quantities of nanoparticles and accordingly, RPV, can be deposited into the skin, then therapeutic plasma levels of the drug could theoretically be maintained for prolonged periods [[19], [20], [21]]. Furthermore, work recently published by our MAP research team indicated the potential for lymphatic uptake of a nanoparticle-derived tracer dye, when delivered from dissolving MAPs into the skin [17]. Sustained delivery of RPV with distribution to one of the predominant sites of HIV replication, namely the secondary lymph nodes, could have profound effects on inhibition of HIV replication [22]. With all of these considerations taken into account, it is anticipated that, in time, the development of an RPV LA MAP, importantly, with the potential for self-administration, could expand access and adherence to HIV treatment, particularly in low-resource settings and could obviate requirements for the use of needles in drug administration.

## 2 Materials and methods

### 2.1 Materials

Poly(vinylpyrrolidone) (PVP) of molecular weight 360,000 Da; poly(vinyl alcohol) (PVA) of molecular weight 9000–10,000 Da; poly(ethylene glycol) (PEG) of varying molecular weights (200–600 Da) and glycerol were all purchased from Sigma-Aldrich, Gillingham, Dorset, UK. Gantrez™ S-97, copolymer of methyl vinyl ether and maleic acid (PMVE/MA), with a molecular weight of 1500,000 Da was a gift from Ashland, Kidderminster, UK. RPV base and long-acting RPV nanosuspension (RPV LA, 300 mg/ml) were supplied by Janssen Pharmaceutical, Beerse, Belgium. Biopsy punches were purchased from Stiefel, Middlesex, UK and Tissue-Tek® optimal cutting temperature medium was obtained from Sakura Thatcham, UK.

### 2.2 Preparation of PVA/RPV LA formulations for MAP casting

**Fig. 2:**
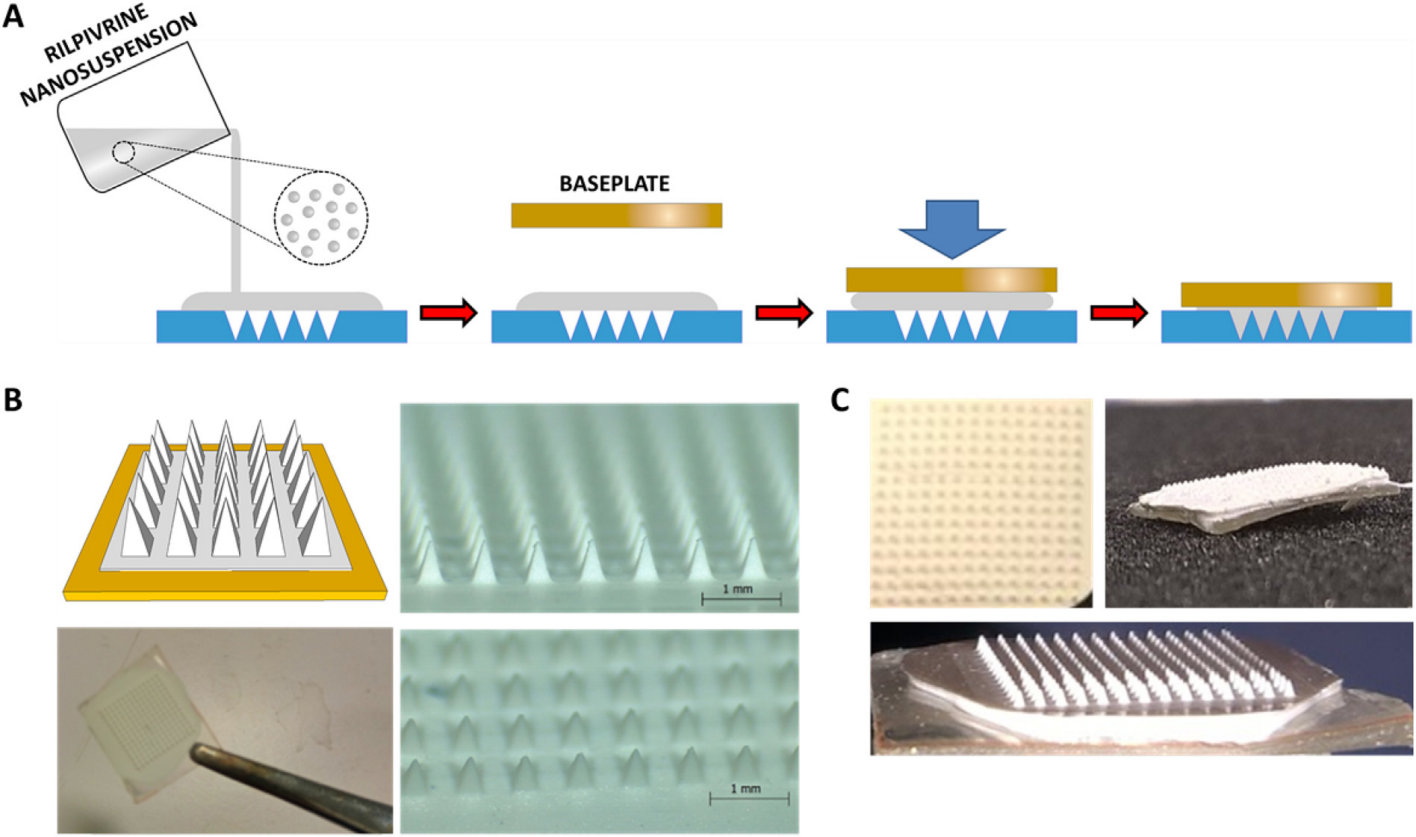
Schematic representation of the RPV LA MAP manufacturing process (A); micrographs of exemplar RPV LA MAPs generated (B) and digital images of RPV LA MAPs (C).

Low molecular PVA (MW 9000–10,000 Da) was used to formulate the microneedles of the arrays and due to the low water solubility of RPV, a long-acting RPV nanosuspension (RPV LA), developed and supplied by Janssen Pharmaceutical, was the drug source used throughout this work. MAPs were prepared using aqueous formulations containing varying amounts of PVA and RPV LA. The formulations were named according to their composition. For example, the 80R10P formulation was cast with 80% (*w*/w) RPV LA and 10% (w/w) PVA, as listed in Table 1. The process by which the MAPs were cast is depicted schematically in Fig. 2A and B. Images of exemplar RPV LA MAPs are presented in Fig. 2C.

**Table 1:** Composition of the formulations used to prepare RPV LA MAPs.

Formulation	Composition (%) (*w*/w)		
	RPV LA	PVA	Water
100R	100	0	0
80R10P	80	10	10
70R15P	70	15	15
60R20P	60	20	20

The baseplate used in all of the documented studies was formulated using aqueous solutions of 20% (w/w) PVP of molecular weight 360,000 Da and 1.5% (*w*/w) glycerol.

### 2.3 Fabrication process of two-layered dissolving RPV LA MAPs

Dissolving RPV LA MAPs with bioadhesive baseplates were prepared in a two-step process of microneedle casting, followed by baseplate adhesion behind the microneedles. The process is illustrated in Fig. 2A. Briefly, silicone MAP moulds were designed in the geometry: 14 microneedles × 14 microneedles with microneedle heights of 600 μm (the archetypal microneedle heights utilized in a vast range of our previously published studies), base widths of 300 μm and interspacing of 300 μm. MAPs were prepared by dispensing 150 mg of casting gel onto the top of the MAP moulds. A preformed, dry baseplate, cast from an aqueous blend containing 20% (*w*/w) PVP and 1.5% (w/w) glycerol, was then placed behind the microneedles. The moulds were placed inside a positive pressure compression chamber and a pressure of 3 bar was applied for 15 min. Finally, the MAPs were dried at room temperature for 24 h and were then removed from the moulds and used in mechanical tests. The RPV LA containing formulation was distributed between the needle tips and a thin baseplate behind the needles.

Different formulations were prepared but it was established that the optimum formulation was that containing 70% (*w*/w) RPV LA, 15% (w/w) PVA and 15% (w/w) water (see results section). These MAPs were produced containing a total of 30 mg of RPV LA. The RPV LA loading in the microneedles was calculated using Eq. 1, considering the pyramidal geometry of the needle tips, the experimentally-determined density of the RPV LA/PVA dry formulation and the calculated concentration of RPV per mg of RPV LA/PVA dry formulation.

(1) $$\mathrm{RPV}\;\mathrm{in the needle tips}=N∙\frac{\left(h∙b^{2}\right)∙\rho ∙\left[\mathrm{RPV}\right]}{3}$$

where N is the number of microneedles per MAP; h and b are height and base width of the microneedles (600 and 300 μm, respectively); ρ is the density of the dry formulation and [RPV] is the concentration of RPV in the dry formulation (expressed in mg RPV/mg material). To obtain the density of the dry formulation, the following equation was used: ρ = w/SX, where X is the mean thickness of the film, S is the cross-sectional area and w is the weight of the film.

### 2.4 Determination of the insertion/mechanical capabilities of RPV LA MAPs

The Parafilm® skin simulant model was employed, as previously described, as a means of determining the mechanical strength of the RPV LA MAPs [[23], [24], [25]].

### 2.5 Evaluation of the effect of PVA on NP size

The effect of PVA on the NP size within the RPV LA was then evaluated. A Nano ZS Zetasizer and DTS software (Malvern Instruments Ltd., Worcestershire, UK), employed at a temperature of 25 °C, was used in these experiments. Two samples, namely, RPV LA diluted in water and RPV LA MAPs that were prepared, dried and then dissolved in water were analysed.

### 2.6 Pharmaceutical analysis of RPV from *ex vivo* samples

RPV quantification from excised neonatal porcine skin samples was performed using a Phenomenex™ Luna 5 μm C18 [2] 100 Å column (150 × 4.6 mm; Phenomenex, Cheshire, UK) held at 30 °C ± 5 °C and a RP-HPLC (Waters Alliance HPLC System (e2695 separation module; Waters Corporation, Milford, UK)) with UV detection at 282 nm. The mobile phase was composed of 60:40; acetonitrile:water, with a flow rate of 1 ml/min and a run time of 10 min per sample. The injection volume was 20 μl. The chromatograms obtained were analysed using Waters Empower® 2 software. Least squares linear regression analysis and correlation analysis were performed on the calibration curves produced, enabling determination of the equation of the line, its coefficient of determination and the residual sum of squares (RSS). The limits of detection (LOD) and quantification (LOQ) for this method, 0.17 μg/ml and 0.55 μg/ml, respectively, were determined using an approach based on the standard deviation of the response and the slope of a representative calibration curve, according to the Q2(R1) guidelines from the International Conference on Harmonisation (ICH) [26]. RPV standards were prepared in mobile phase.

### 2.7 MAP in-skin dissolution

The dissolution rate of the RPV LA MAPs was investigated in excised neonatal porcine skin. Skin samples were carefully shaved using a disposable razor and then equilibrated in PBS (pH 7.4) before being mounted onto a styrofoam block, coated with PBS-soaked absorbent paper. MAPs were inserted into the skin with a pressure of 10 N/MAP over 30 s using a TA.XTPlus Texture Analyser (Stable Micro Systems, Surrey, UK) and the skin was then stored at 37 °C for the duration of the experiment. MAPS were removed at designated time points and digitally imaged. Optical coherence tomography (OCT) (Michelson Diagnostics Ltd., Kent, UK) was used to assess the successful insertion of the MAPs into the excised skin, as reported previously [16,23].

### 2.8 Delivery of RPV LA into full-thickness porcine skin

Full-thickness skin, collected from stillborn piglets, was shaved using a razor and then mounted onto a styrofoam block, coated with PBS-soaked absorbent paper. RPV LA MAPs were inserted into the skin using manual force, roughly equivalent to 20 N, over 30 s [23,24]. The MAPs were maintained in place for 30, 60 or 120 min and the samples were incubated at 37 °C over the course of the experiment to mimic *in vivo* conditions. Subsequently, the RPV LA MAPs were removed and a tissue sample, taken from that portion of the skin where the RPV LA MAP had been inserted, was obtained using a biopsy punch (6 mm diameter) (Stiefel, Middlesex, UK). A diagrammatic representation of this procedure is presented in Fig. 5A. The samples were frozen, mounted, ventral surface facing upwards, using optimal cutting temperature medium (Sakura® Thatcham, UK) and cut into 50 μm sections using a Leica CM1900 Cryostat (Leica Microsystems UK Ltd). To analyse the amount of RPV LA that permeated into each slice, mobile phase was added to tissue sections and these were vortexed for 20 min to solubilise the drug. Samples were then centrifuged at 14,000× *g*, 10 min. Supernatants were analysed using the validated HPLC methodology.

### 2.9 Pharmaceutical analysis of RPV from *in vivo* samples

RPV quantification from rat plasma was performed using a Phenomenex SphereClone 5 μm (150 × 4.60 mm) column (Phenomenex, Cheshire, UK) and a RP-HPLC (Agilent 1200® Binary Pump, Agilent 1200® Standard Autosampler, Agilent 1200® Variable Wavelength Detector, Agilent Technologies UK Ltd., Stockport, UK) with UV detection at 282 nm. The mobile phase was composed of 65:35; acetonitrile (ACN):water containing 0.1% (*v*/v) trifluoroacetic acid (≥ 99% pure TFA), with a flow rate of 1 ml/min, and a run time of 10 min. The injection volume was 20 μl. The chromatograms obtained were analysed using Agilent ChemStation® Software B.02.01. Least squares linear regression analysis and correlation analysis were performed on the calibration curves produced, enabling determination of the equation of the line, its coefficient of determination and the residual sum of squares (RSS). The limits of detection (LOD) and quantification (LOQ) for this method were estimated according to the signal-to-noise ratio guidelines outlined in the European Pharmacopoeia, version 8.0 [27]. LOD and LOQ ratios of 3:1 and 10:1, respectively, were used throughout the analysis. RPV standards were spiked into blank rat plasma and standards of 50 ng/ml met the LOQ of the system. For a calculation of the mean plasma concentrations, samples below the LOQ were treated as half the LOQ (*i.e.* 25 ng/ml).

### 2.10 Extraction of plasma and drug

The extraction of RPV from spiked, control plasma and test plasma from rats in the MAP or IM cohorts was carried out as described previously [[28], [29], [30]].

### 2.11 In vivo studies

#### 2.11.1 Application of RPV LA MAPs

Ethical permission for all *in vivo* experiments was obtained from the Queen's University Belfast, Biological Services Unit (BSU) and all researchers carrying out the work held Personal Licences from the UK Home Office. Two cohorts were employed in *in vivo* studies: the intradermal (MAP) cohort and the control IM (IM) cohort. Experiments were conducted using female Sprague Dawley rats aged between 9 and 13 weeks upon commencement of the study and aged between 14 and 17 weeks upon completion of the study. Prior to the commencement of experiments, rats were acclimatised to laboratory conditions for a 7-day period. MAP applications were carried out as described previously and MAPs were removed after 24 h [29]. An image of an exemplar MAP post-application for 24 h is presented in Fig. 5A.

Those rats in the IM cohort received 60 μl of 30 mg/ml RPV LA (1.8 mg dose) into the right hind thigh muscle.

#### 2.11.2 Quantification of RPV in blood and vaginal wash samples from treated animals

Blood samples were collected from the treated rats, at pre-determined time points (1, 4, 7, 28 and 56 days), into heparinised tubes *via* the lateral tail vein. Plasma was separated from the whole blood and prepared as described, prior to HPLC analysis.

Vaginal washes were collected by washing the vaginal cavity with 200 μl sterile water for injection (B. Braun Medical Ltd., Sheffield, UK) at pre-determined time points (1, 4, 7, 28 and 56 days). The samples were centrifuged at 14,000× *g* for 10 min at 4 °C to remove any debris. A 35 μl volume of sample was then mixed with 65 μl acetonitrile (ACN), and a sample of this was analysed by HPLC. For data analysis purposes, arithmetic means were determined and plasma samples with RPV concentrations below the LOQ of the system were included in data tables as half the LOQ, namely 25 ng/ml.

#### 2.11.3 Quantification of RPV in vaginal tissue and lymph nodes of treated animals

Animals were culled at the pre-determined experimental endpoints by CO_2_ overdose and vaginal tissue, axillary, iliac and external lumbar lymph nodes were excised. The lymph nodes were weighed and then homogenised at 50 Hz for 10 min in a Qiagen TissueLyser LT (UK Qiagen Ltd. Manchester, UK). Samples were centrifuged at 14,000× *g* for 10 min at 4 °C and supernatants were collected. The nodes were then subjected to a second extraction procedure. Supernatants were dried under a stream of nitrogen at 37 °C for 50 min, samples were reconstituted in mobile phase and RPV content was quantified using the validated HPLC methodology. Vaginal tissue was treated similarly; however, the tissue was disrupted mechanically prior to homogenisation to increase the surface area in contact with the ACN, maximizing drug recovery. The amount of RPV in the organs was expressed as ng of drug per g of tissue (ng/g), in order to account for differences in tissue masses between rats. For data analysis purposes, arithmetic means were determined and plasma samples with RPV concentrations below the LOQ of the system were included in data tables as half the LOQ, namely 25 ng/ml.

#### 2.11.4 Estimation of human dose and patch size

Estimation of human dose for RPV LA MAP were conducted to provide the same trough concentration as that observed after single-dose RPV LA IM, 600 mg, after 28 days (44 ng/ml), as this is the dosing regimen and monthly therapeutic RPV LA IM regimen in clinical development [29]. Given the current study evaluated single-dose RPV LA MAP and RPV LA IM, the exposure achieved after single-dose administration of RPV LA IM in humans was selected rather than trough concentrations achieved at steady-state. Using the mean concentration-time data, the relative ratio of exposures after adjusting for differences in doses administered between MAP and IM administration were estimated. The relative ratio of exposures at the various time points after dosing were utilized to estimate dose of RPV LA MAP needed to achieve 44 ng/ml using the human observed RPV LA IM data from Jackson 2014 as the comparator [29].

### 2.12 Statistical analysis

Data was analysed using the One-Way ANOVA parametric test, followed by Tukey's multiple comparison post-hoc test. A *p* value <0.05 was denoted as a significant difference.

## 3 Results and discussion

### 3.1 Preparation of various PVA/RPV LA formulations for MAP casting

MAPs were manufactured containing two layers. RPV LA was incorporated into the needle tips and into a thin film behind the needles. A rigid polymeric baseplate was added to facilitate insertion. Multiple published studies have previously used multi-layered MAPs [16,25,[31], [32], [33]]. The novelty of the present work was not the use of a multi-layered MAP, but rather the exploratory nature of this approach to evaluate the potential application of MAPs in delivery of long acting formulations, and consequently to maximize the amount of RPV LA administered. Previous studies have suggested that dissolving MAPs are capable of delivering not only the cargo located in the needle tips but also, in some instances, drug in the baseplate [29]. Consequently, these MAPs contained a thin film containing RPV LA formulation behind the needle tips, to maximize the amount of formulation delivered.

**Fig. 3:**
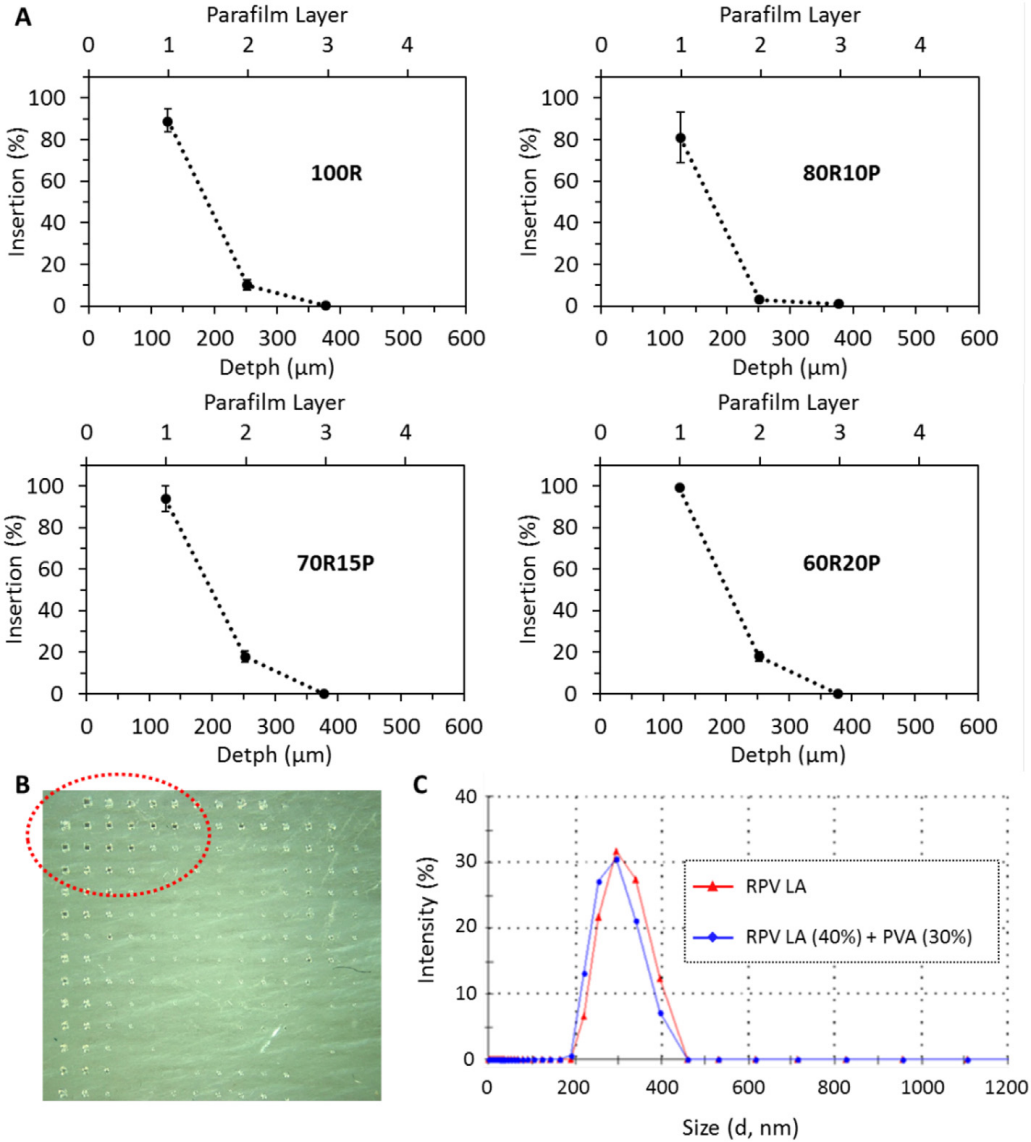
Insertion profile of RPV LA MAPs containing different amounts of PVA and RPV LA into a Parafilm® multi-layered skin simulant, following the application of a pressure of 10 N/MAP, (means ± SD, *n* = 3) (A). Digital image of the first layer of the skin simulant after the insertion of MAP formulated containing no PVA (B). Size of RPV nanoparticles in nanosuspension (RPV Nanosusp) and RPV nanoparticles following MAP formulation with PVA, drying and then dissolution of the MAP in water (RPV Nanosusp (40%) + PVA (30%)) (C).

MAPs were prepared using RPV LA formulated from aqueous blends containing various PVA concentrations ranging from 0%–20% (*w*/w) and baseplates cast from aqueous blends of 20% (w/w) poly(vinylpyrrolidone) (PVP), 1.5% (w/w) glycerol. The Parafilm® skin simulant model was employed, as previously described, as a means of determining the insertion ability of the RPV LA MAPs [23,24]. Upon application of a pressure of 10 N/MAP over 30 s using a TA.XTPlus Texture Analyser (Stable Micro Systems, Surrey, UK), the profiles of the various MAPs indicated that they had sufficient mechanical strength to successfully insert into the Parafilm® multi-layered skin simulant (Fig. 3A). Regardless of the formulation employed to cast the microneedles, the MAPs consistently pierced through two layers of Parafilm®, indicating constant and reproducible depths of insertion (Fig. 3A). MAPs cast from aqueous blends containing <15% (*w*/w) PVA were brittle, however, and some microneedles broke off the arrays upon insertion, indicating a lack of mechanical strength.

### 3.2 Dissolving RPV LA MAP composition

The dissolving RPV LA MAPs were fabricated in two stages to avoid drug wastage (Fig. 2). Following formulation optimisation studies, the RPV LA MAPs used in all subsequent experiments were cast from aqueous blends of 70R15P in the MAP and 20% (*w*/w) PVP, 1.5% (w/w) glycerol in the baseplates. The MAPs were prepared as outlined schematically in Fig. 2A. The actual loadings of RPV LA into the RPV LA MAP equated to 30 mg in patch, of which 2 mg (1.9 ± 0.2 mg) was in the microneedles of the arrays.

### 3.3 Determination of the insertion/mechanical capabilities of RPV LA MAPs

The percentage of holes created in Parafilm® layers for the different dissolving MAP formulations tested is shown in Fig. 3A. The *in vitro* insertion depths obtained for the four formulations displayed similar trends and all were capable of piercing three layers of Parafilm®. The mean thickness of a Parafilm® layer is 126 ± 7 μm [23], thus suggesting that the MAPs inserted to depths of at least 378 μm of the total 600 μm height of the microneedles. The results generated in this study, therefore, are consistent with work we have previously published using a variety of other MAP formulations, where the insertion was estimated to be approximately 60% of the total microneedle length [23,25]. MAPs containing no PVA but rather 100% RPV LA, although able to penetrate through the Parafilm® layers, did not have sufficient mechanical strength, as some of the microneedles broke away from the arrays and lodged in the layers of Parafilm®, as indicated in Fig. 3B. The MAPs formulated with 70R15P showed superior mechanical stability compared to the MAPs containing 10% (*w*/w) PVA and results indicated that they could be inserted deeper into skin layers. Consequently, the 70R15P formulation was selected as the optimal formulation for MAP preparation.

### 3.4 Evaluation of the effect of PVA on NP size

The properties of the nanosuspension should not be modified after being combined with PVA or dried to form the MAPs. Consequently, the particle size was evaluated before and after the formulation process. Even at the highest loading of PVA, there was no significant effect on RPV nanoparticle size or polydispersity (Fig. 3C). It was apparent that after formulating RPV LA in combination with PVA, the MAPs could be easily dissolved in water and the RPV LA particle size distribution did not change and continued to be monodisperse. Particle size distribution is an important attribute for intradermal drug delivery [17]. Therefore, it was crucial to ensure that the physicochemical properties of the nanosuspension did not change post-formulation. Importantly, the results obtained here suggest that there was no significant interaction between the PVA and the RPV LA, thus suggesting that no modification of the particle occurred upon formulation and fabrication of the MAPs.

### 3.5 MAP in-skin dissolution

**Fig. 4:**
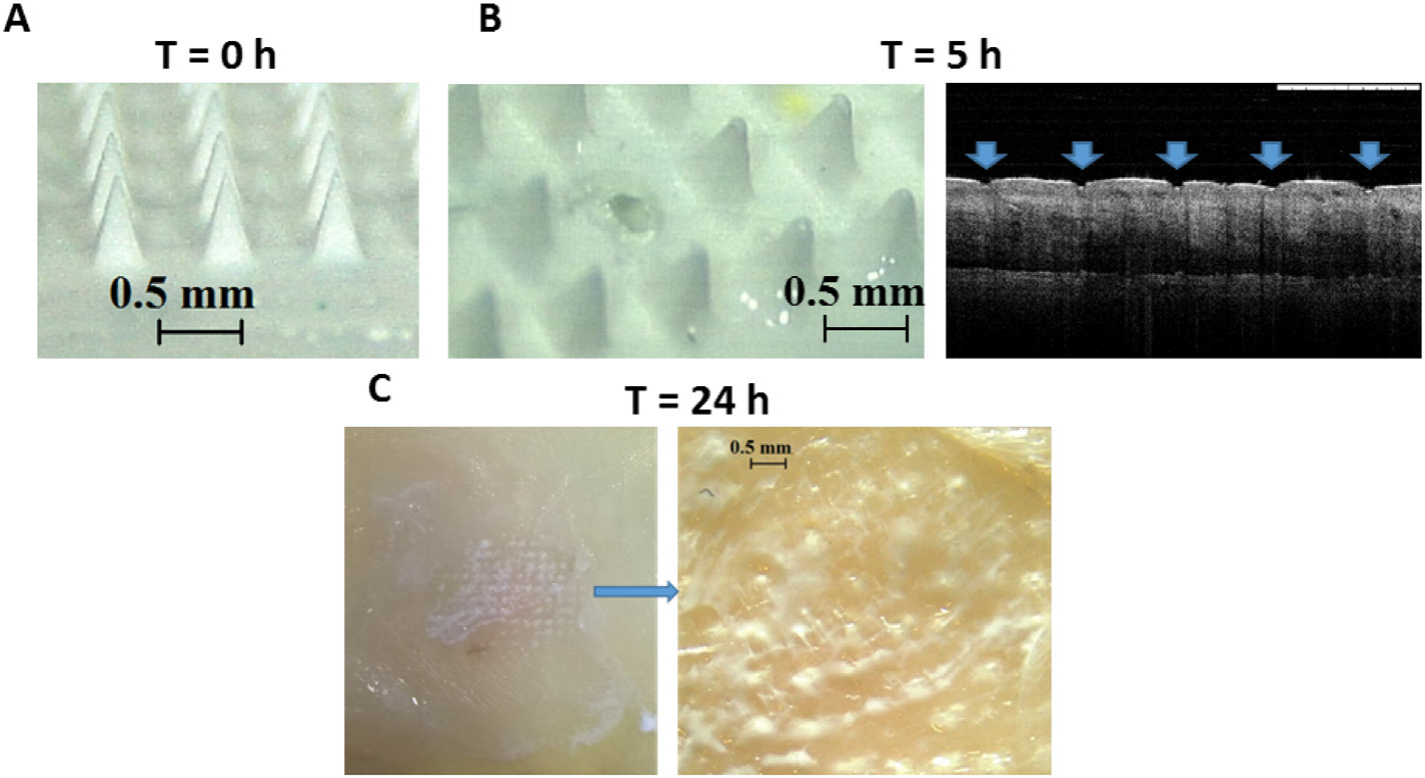
Representative digital micrographs illustrative of the dissolution of RPV LA MAP at specific time points (0, 5 and 24 h) in excised neonatal porcine skin. Digital micrograph of a RPV LA MAP prior to insertion into skin (A). Digital micrograph of a RPV LA MAP upon removal from the skin after an insertion time of 5 h and OCT image of the resulting microconduits in the porcine skin following MAP removal (B). Digital image and micrograph of porcine skin upon removal of RPV LA MAP post-24 h insertion with RPV LA visible in the microconduits which were created in the skin (C).

The dissolution of the needles of the RPV LA MAP in excised skin occurred slowly, likely due to the high content of hydrophobic RPV LA in the needles of the MAPs. The microscopic images presented in Fig. 4A and B illustrates the dissolution of the needles. After 5 h, the needles are no longer sharp and some have detached from the MAP, remaining *in situ* in the skin. The pores created in the skin, 5 h post-MAP application are clearly defined in the OCT image presented in Fig. 4B. Following removal of the RPV LA MAP from the skin at 24 h, RPV LA deposits are clearly visible in the skin (Fig. 4C).

### 3.6 Delivery of RPV into full-thickness porcine skin

**Fig. 5:**
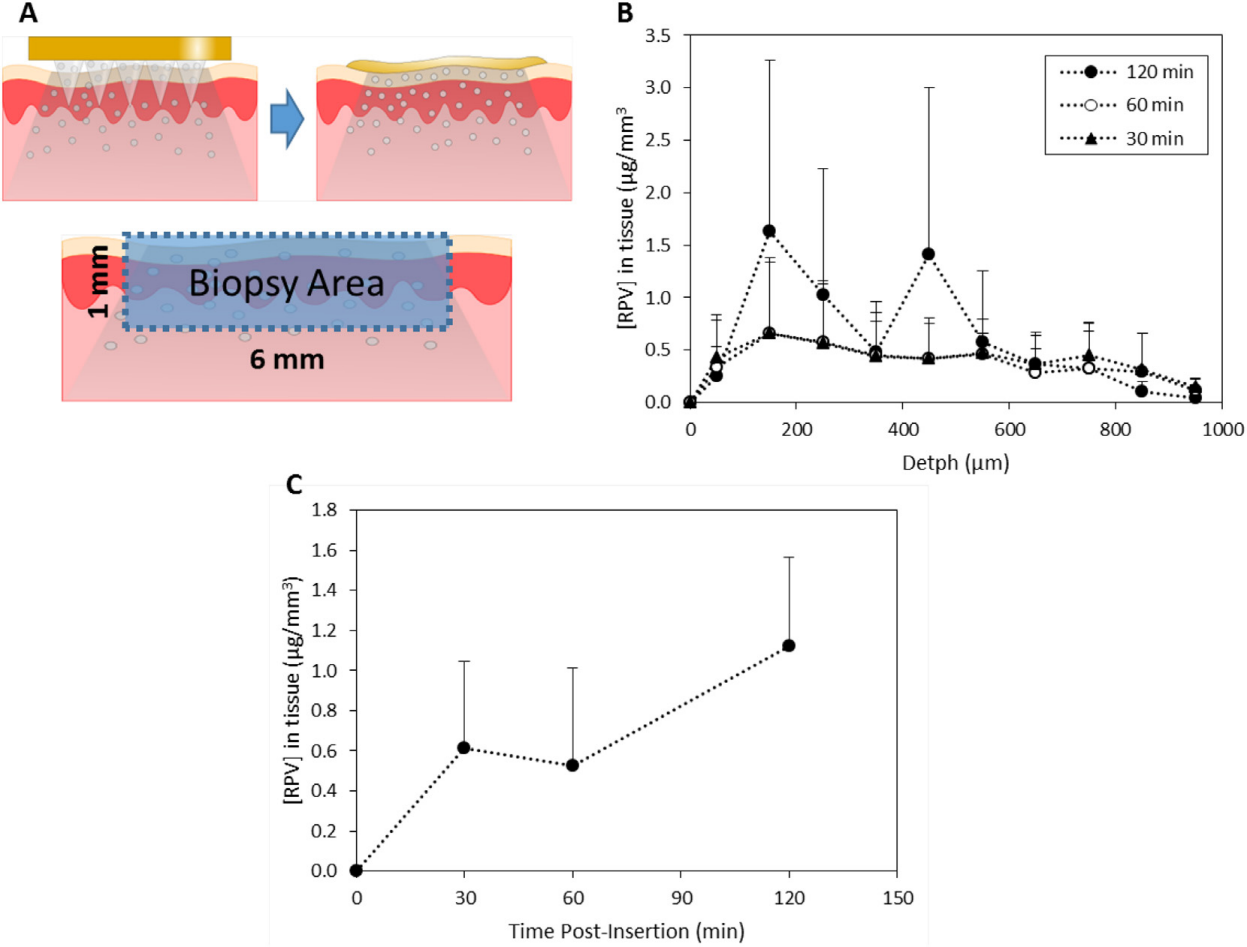
Insertion of RPV LA MAP into excised porcine skin, followed by deposition studies. RPV LA MAPs were inserted, for three different durations (30, 60 or 120 min), into excised full-thickness neonatal porcine skin, and following this, the deposition of drug into a biopsied piece of skin was determined. Schematic representation of the experimental approach, indicating diffusion of drug into the excised skin (A). RPV concentrations (μg/mm^3^) in excised neonatal porcine skin were determined, at the relevant time points, and as a function of the depth of the tissue sections. This experiment was carried out under temperature control at 37 °C, (means ± SD, *n* = 3) (B). The total amount of RPV (μg/mm^3^) in the biopsied tissue was expressed as a function of the application time (means ± SD, n = 3) (C).

The results presented in Fig. 5 depict the amount of RPV, in relation to skin depth, detected per mm^3^ of tissue, across three different application times, namely 30, 60 and 120 min (Fig. 5A, B). The concentration of RPV in the tissue increased as a function of the application time in the initial approximately 200 μm of tissue (Fig. 5B). The drug concentrations, in samples from the 30 and 60 min application durations, levelled off and remained relatively constant between approximately 200 and 600 μm, after which concentrations began to decrease. There was more variation in the RPV concentrations detected in samples from the 120 min application. In all instances however, RPV was detected in skin sections at depths >600 μm, therefore deeper in the skin layers than the heights of the MAPs themselves, indicating movement of the drug in the tissue. Specifically, the total amounts of RPV detected in the tissue sections at each of the time points were determined and are presented in Fig. 5C. There is a marked increase in the RPV present in the tissue after 120 min. This increase is possibly due to the slow solubilisation of the needles containing RPV LA. The diffusivity of nanoparticles is likely to be less than that of solubilised drug and, additionally the hydrophobic nature of RPV LA would lead to slower dissolution of the needles [34], as indicated by in-skin dissolution studies.

### 3.7 Quantification of RPV in plasma and vaginal wash samples from treated animals

**Fig. 6:**
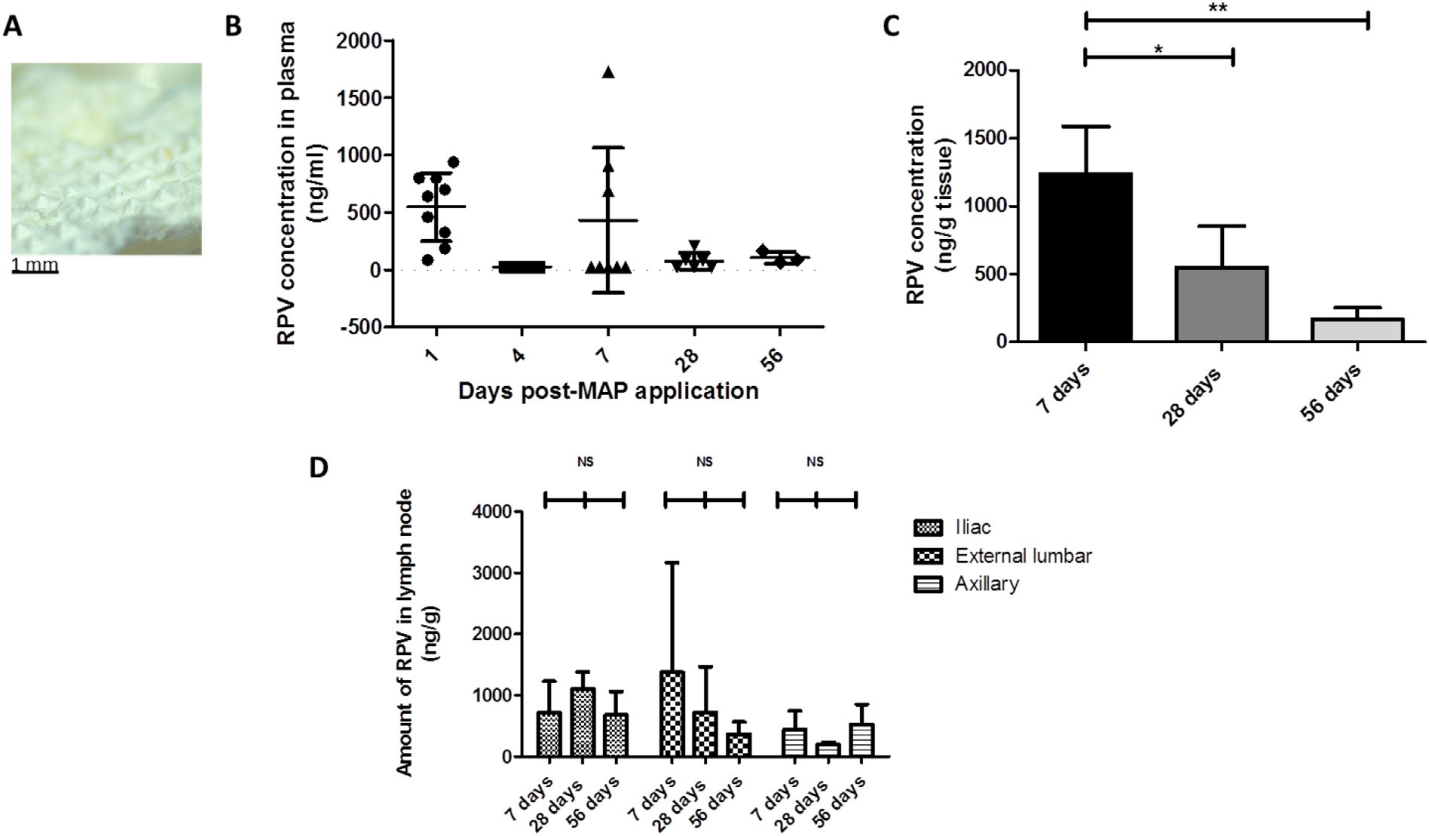
RPV quantification in *in vivo* samples from animals treated with 4 × RPV LA MAPs (4 × 2 mg RPV LA) (MAP cohort, three rats for each time point). An exemplar MAP post-removal from a rat, indicating the extent of MAP dissolution over the course of the 24 h application period (A). Plasma levels of RPV at 1, 4, 7, 28 and 56 days post-MAP application, (means ± SD, *n* ≥ 3 at each time point, in accordance with the experimental regime employed) (B). No plasma at the 4 day time point had RPV levels above the LOQ of the system and so for analysis purposes, samples were treated as 25 ng/ml. Determination of the amount of RPV in excised vaginal tissue, expressed as ng of drug per g of vaginal tissue (ng/g), (means ± SD, *n* = 3; * *P* < 0.05; NS = not significant) (C). Determination of the amount of RPV in excised lymph nodes, expressed as ng of drug per g of lymph node tissue (ng/g) in animals treated for 7, 28 or 56 days, (means ± SD, n = 3 in all cases; NS = not significant) (D). No axillary nodes at the 28-day time point or external lumbar nodes at the 56-day time point had RPV levels above the LOQ of the system. For analysis purposes, these samples were all treated as 25 ng/ml and then adjusted for tissue mass.

Female rats were treated with either an IM dose of RPV LA (1.8 mg) (four rats) or with simultaneous application of 4 × MAPs (of which 2 mg RPV LA/array was contained in the microneedles) (three rats). In the MAP cohort, MAPs were applied to the backs of the rats and removed after 24 h. It is evident from the exemplar digital image of a single MAP post-removal (Fig. 6A) that not all MAPs dissolved over the duration of application, which is not surprising as these MAPs have a high content of poorly soluble particles. This finding is consistent with the *ex vivo* results reported in the previous section. Accordingly, not all drug encapsulated within the MAPs would be expected to be delivered over the course of the 24 h application time, unlike the complete delivery from the IM injections. Importantly, there was no indication of irritation on the skin of the rats following MAP application and removal.

**Fig. 7:**
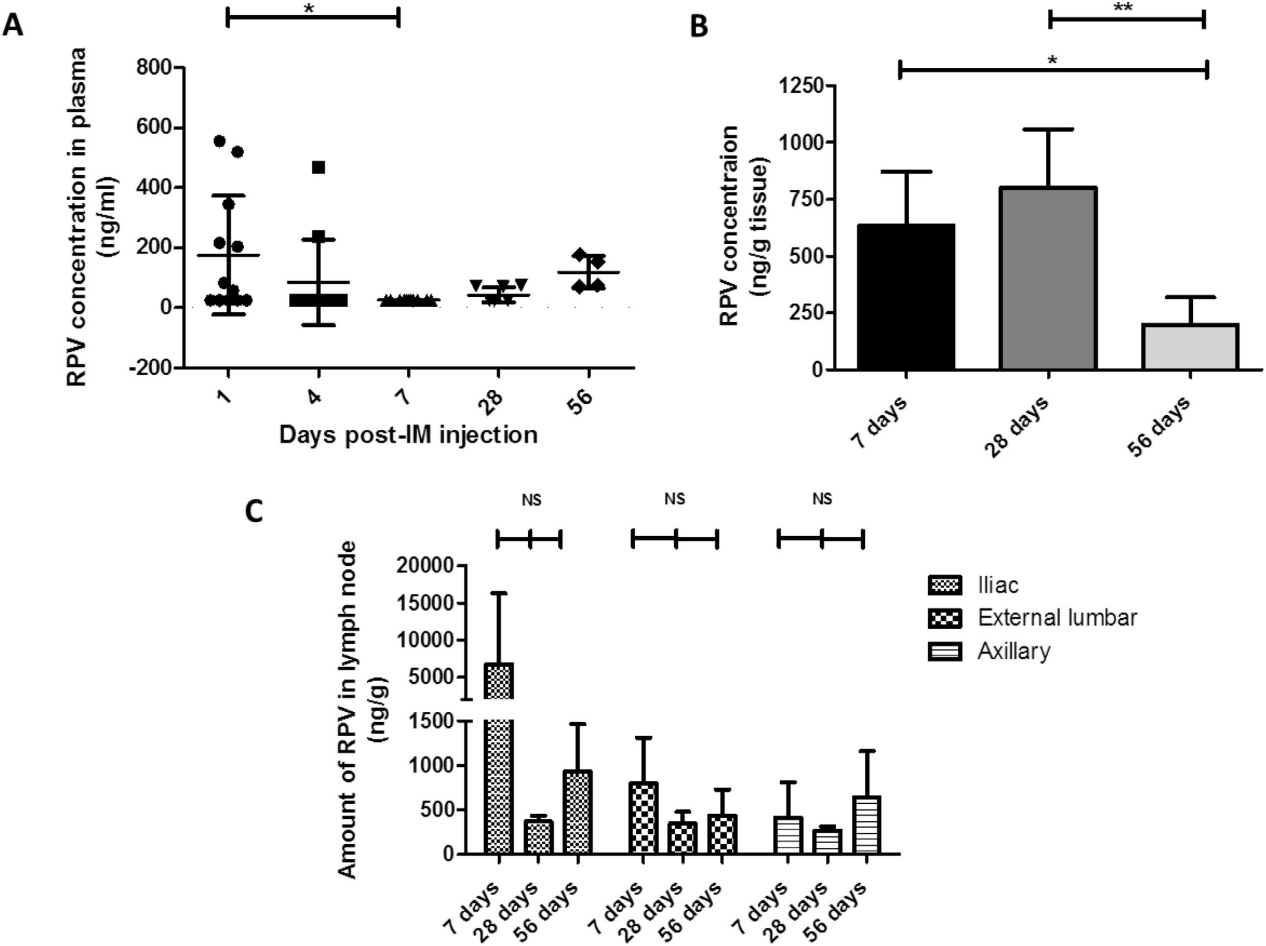
RPV quantification in *in vivo* samples from animals treated with RPV LA *via* IM injection (1.8 mg) (IM cohort, four rats for each time point). Plasma levels of RPV at 1, 4, 7, 28 and 56 days post-IM injection of RPV, (means ± SD, *n* ≥ 4 at each time point, in accordance with the experimental regime employed) (A). No plasma at the 7-day time point had RPV levels above the LOQ of the system and so for analysis purposes, samples were treated as 25 ng/ml. Determination of the amount of RPV in excised vaginal tissue, expressed as ng of drug per g of vaginal tissue (ng/g), (means ± SD, *n* = 4; * *P* < 0.05; NS = not significant) (B). Determination of the amount of RPV in excised lymph nodes, expressed as ng of drug per g of lymph node tissue (ng/g) in animals treated for 7, 28 or 56 days, (means ± SD, n = 4 in all cases; NS = not significant) (C). No lymph nodes at the 28-day time point had RPV levels above the LOQ of the system. For analysis purposes, these samples were all treated as 25 ng/ml and then adjusted for tissue mass.

Plasma samples were collected from the animals after MAP and IM administration and the mean concentrations of RPV in each cohort, at sampling time points of 1, 4, 7, 28 and 56 days, are presented in Figs. 6B and 7A, respectively. Those rats treated with RPV LA MAP exhibited high RPV plasma concentrations at 1 and 7 days post-MAP application (Table 2).

**Table 2:** Mean plasma RPV concentrations and data ranges after MAP (8 mg) and IM (1.8 mg) administration of RPV in rats.

Time (Day)	MAP mean plasma concentrations and ranges (ng/ml)	n	IM mean plasma concentrations and ranges (ng/ml)	n
0	0.0	9	0.0	12
1	547.5 (85, 939.3)	9	175.5 (NQ, 555.8)	12
4	<LLOQ	6 (3 sample errors)	84.7 (NQ, 468.4)	11 (1 sample error)
7	430.7 (NQ, 1725.7)	9	<LLOQ (NQ)	11 (1 sample error)
28	75.6 (NQ, 208.7)	6	42.9 (NQ, 75.2)	8
56	104.4 (63.1, 162.6)	3	118.9 (70.4, 177.2)	4

Plasma concentrations below the lower limit of quantification (LLOQ), or not quantifiable (NQ), were treated as 25 ng/ml in analyses (half the lower limit of quantification).

Specifically, the maximum mean plasma concentration of 547.5 ± 298.1 ng/ml was recorded just 1 day post-MAP application with 430.7 ± 608.1 ng/ml detected after 7 days. At the Day 4 time point, however, no samples within this cohort had RPV concentrations above the limit of quantification (LOQ) of the system. This was not unexpected, however, as the RPV detected 1 day post-MAP application would have consisted of rapidly dissolving RPV delivered intradermally *via* MAP that had entered the systemic circulation. Four days post-MAP application, encapsulated RPV could have accounted for the majority of the drug in the animal and this would not have been quantifiable in the plasma, as the drug would not yet have reached the systemic circulation. Plasma concentrations of RPV subsequently decreased to 75.6 ± 71.5 ng/ml at 28 days and comparable RPV concentrations were maintained up to the experimental endpoint of 56 days (104.4 ± 51.9 ng/ml). Those rats treated with RPV LA *via* IM injection exhibited lower initial RPV plasma concentrations. Following IM injection, a peak concentration of 175.5 ± 197.6 ng/ml was detected 1 day post-injection and no samples had RPV concentrations above the LOQ of the HPLC system at 7 days (25 ± 7.2 ng/ml). Interestingly, the mean RPV plasma concentration at the experimental endpoint of 56 days (118.9 ± 54.2 ng/ml) was comparable to that achieved *via* RPV LA MAP at the same time point (104.4 ± 51.9 ng/ml). In terms of direct comparison of the results generated here with other experimental studies, this is challenging, as stated previously, due to the fact that not all of the drug encapsulated into the MAPs would effectively be delivered *in vivo*. Some preclinical work carried out in 2010 studied the pharmacokinetics, distribution and tolerability of RPV LA delivered *via* IM injection in rats and dogs [35], although the RPV LA used in the earlier work was of lower concentration (2.5%, compared to 30% in the present study). In the 2010 study, the plasma concentrations of the drug increased gradually up to 7 h post-dose and then declined, but remained detectable up to 8 days post-dose, when 5 mg/kg drug was administered, and up to 42 days post-dose when 20 mg/kg was administered. For comparison, as the rats within the IM cohort of the study presented here had a mean starting weight of 242 g, they therefore had the equivalent of approximately 7 mg/kg RPV LA administered. The MAP cohort, with a mean starting weight of 248 g, had the equivalent of approximately 32 mg/kg RPV LA administered in the microneedles of the arrays but again, not all of this would have been delivered. Therefore, across the two studies (present study and the 2010 preclinical study [35]), and considering the amount of drug administered, rats treated *via* IM or MAP exhibited comparable patterns of sustained drug in plasma over similar time frames. In the 2010 study, those rats given 5 mg/kg and 20 mg/kg RPV LA had *C*_max_ of 71 ng/ml and 158 ng/ml, respectively at 7 h post-dose [35]. In the present study, mean plasma concentrations of approximately 176 ng/ml (IM cohort) and 548 ng/ml (MAP cohort) were recorded at 24 h, but no blood samples were collected earlier. The higher plasma concentrations of drug detected in this study, and at a later time point, may be due to the inherent differences in the pharmacokinetic profiles for the drug, as determined by the differences in RPV concentration between the two formulations. As there are no previous studies that have delivered this or any other LA nanosuspension drug in rats *via* MAP, the plasma concentrations of drug post-MAP delivery of RPV LA must be considered in comparison to the IM control. In this regard, at 24 h, plasma levels of the drug in the MAP cohort (547.5 ± 298.1 ng/ml) exceeded those of the IM cohort (175.5 ± 197.6 ng/ml). When the differences in RPV LA dose administered are taken into consideration, this shows the potential intradermal delivery capabilities of this nanosuspension drug from MAPs. As stated earlier in the discussion, this assertion is further backed up by the fact that the two delivery routes resulted in equivalent mean plasma levels of the drug in rats at the experimental endpoint of 56 days.

There is currently no consensus therapeutic plasma concentration for RPV but the documented drug plasma level above the protein-binding adjusted concentration required to inhibit 90% of *in vitro* viral replication (IC90) of 12.5 ng/ml has been suggested previously [5]. With this in mind, future studies must be carried out to optimise and lower the LOQ, in order to improve the pharmacokinetic measurements. As a precursor to this and using the data generated in this study, and comparing to the plasma RPV levels determined by Jackson and colleagues in a clinical pharmacokinetic study of IM RPV LA [28], we can very cautiously predict the MAP patch size that may be necessary for use in human studies. RPV LA has conventionally been administered at monthly intervals *via* IM injection. Accordingly, convention dictates that an optimised MAP would be anticipated to also deliver RPV LA for 28-day exposure. RPV LA IM at a dosing regimen of 600 mg every 4 weeks is in Phase 3 clinical development for HIV prevention. The 2014 clinical study, carried out in HIV-negative volunteers, demonstrated that 600 mg IM dosing of RPV LA provided a mean plasma concentration of 44.2 ng/ml, 28-days after single-dose administration [28]. By evaluating the relative exposures between MAP and IM administration and adjusting for differences in doses administered between the formulations used in this study, the prior published clinical data was used to estimate the dose of RPV LA required for delivery by MAP. Based upon the 28 day trough concentrations achieved after single-dose administration of 600 mg IM RPV LA, an RPV LA dose in the RPV LA MAP of 1500 mg, equating to a patch size of approximately 375 cm^2^, is estimated to be needed to achieve similar duration of exposure after single-dose administration. A patch of this size would be unrealistic and inappropriate for patient usage however. Theoretically, an RPV LA MAP of approximately 28 cm^2^ could be cautiously predicted to maintain therapeutic concentrations of RPV in plasma over 7 days in humans, although further *in vivo* pharmacokinetic evaluations will now have to be carried out to bolster this proof of concept data. A patch of 28 cm^2^ would actually be smaller than commercially available transdermal patches including GlaxoSmithKline's (GSK) Nicotinell® nicotine patches of 30 cm^2^ [36] and Janssen's Duragesic® CII (fentanyl) patches of 32 and 42 cm^2^ [37]. In addition, a recent healthy volunteer study demonstrated successful self-insertion of a 16 cm^2^ MAP, following appropriate instruction, demonstrating clear feasibility of applying larger MAP patches [38]. Important to consider is the empowerment such a self-applied and discreet device would offer to individuals, particularly women, in the developing world who, instead of having to go to distant health clinics once a month for LA injections, could apply MAPs at home, thus limiting disruption to their family life and caring responsibilities. Other medicines for chronic conditions are also given at 7-day intervals, such as methotrexate for rheumatoid and juvenile arthritis. The MAP microneedle lengths and densities can be increased with a view to maximizing drug loading into the MAP, thus increasing plasma concentrations and dosing intervals with RPV LA MAP. Future studies are necessary to investigate this.

With reference to RPV in vaginal washes, no RPV was detected in the vaginal washes collected from rats 7, 28 or 56 days after administration of MAP or IM RPV LA. This is in contrast to clinical studies where RPV concentrations in vaginal fluid approximated those seen in plasma [28]. This was potentially caused by low wash volumes or limitations associated with analytical method sensitivity in our preclinical studies.

### 3.8 Quantification of RPV in vaginal tissue and lymph nodes of treated animals

Vaginal tissue was extracted from all animals at the requisite experimental endpoints and RPV concentrations were expressed relative to the mass of the tissue removed from each animal (ng/g tissue). In animals treated with RPV LA MAP, RPV concentrations were highest in animals culled 7 days post-MAP application (1238 ± 347 ng/g tissue) (Fig. 6C) and decreased in rats culled 28 (549 ± 304 ng/g tissue) and 56 (169 ± 84 ng/g tissue) days post-MAP application (Fig. 6C), as anticipated. RPV concentrations in extracted vaginal tissue from rats culled at 7 and 56 days were significantly different (*P* = 0.0273).

In animals treated with RPV LA IM injection, the RPV concentrations in the vaginal tissue did not peak as high as those recorded in the MAP study arm (Fig. 7B). RPV concentrations in the 7 and 28 day cohorts were comparable at 633 ± 239 ng/g tissue and 800 ± 257 ng/g tissue, respectively. The only significant difference in RPV concentrations in the vaginal tissue in this experimental cohort was between 28 and 56 days (*P* = 0.0183). The concentrations in the 56-day IM cohort (197 ± 120 ng/g tissue) were similar to those determined at the same time point in the MAP study arm (169 ± 84 ng/g tissue). Once again, these results show that RPV LA can be successfully delivered intradermally *via* MAP. Interestingly, the localised tissue concentrations of RPV, when delivered from dissolving MAPs, were comparable to those reached *via* conventional IM delivery of the drug. The differences in RPV concentrations in vaginal tissue across the two treatment groups were also compared at each time point and there were no statistically significant differences between the treatment cohorts at common time points.

Secondary lymph nodes are one of the principal sites of HIV replication and the restricted penetration of ARV drugs into these reservoir compartments may be one of the main mechanisms of viral persistence in individuals [22]. Consequently, delivery systems that can target ARV drugs to these compartments are of particular interest and importance. It has been postulated that intradermal injection may also promote enhanced lymphatic uptake when compared to IM or subcutaneous injection. This may be due to higher interstitial pressure and lymph flow rates in the skin relative to other interstitial sites [39]. Accordingly, lymph node–derived concentrations of RPV were also determined in this study. Axillary lymph nodes were extracted as examples of somatic nodes, draining from the skin and underlying musculature, while iliac and external lumbar nodes were extracted as examples of visceral nodes, which drain from the thoracic, abdominal and pelvic organs. The concentrations of RPV in iliac, external lumbar and axillary lymph nodes were then determined and expressed relative to the mass of the tissue removed from each animal (ng/g tissue) (Figs. 6D and 7C). Those rats with RPV levels below the LOQ of the system were treated in data analysis as having RPV of 25 ng/ml, which was then adjusted for the tissue mass of individual nodes.

In the MAP arm of the study, two out of three rats in the 7-day cohort had quantifiable RPV in the iliac nodes, external lumbar nodes and axillary nodes. In the 28- and 56-day cohorts, however, only the iliac nodes had quantifiable RPV in *all* of the animals, with concentrations of 1106 ± 278 ng/g tissue and 679 ± 382 ng/g tissue for the 28- and 56-day cohorts, respectively. RPV concentrations in the axillary nodes at 28 days and the external lumbar nodes at 56 days were all below the LOQ of the HPLC method. Irrespective of the cohort and as such, the length of time post-RPV LA MAP application, RPV was consistently found in the iliac nodes of all animals treated *via* these dissolving MAPs. The detection of RPV in lymph nodes over extended periods draws a parallel with a recent study using a long-acting ARV drug-combination which reported the same phenomenon [40]. In the MAP arm of the present study, there were no significant differences in RPV concentrations in any of the lymph nodes across the different time points.

In contrast to the MAP study arm, after IM administration RPV was not quantified in the iliac nodes of all of the animals employed in the study. Rather, RPV concentrations were below the LOQ of the HPLC system in the iliac nodes of all of the animals in the 28-day cohort, with only one animal in the 56-day cohort having quantifiable RPV (Fig. 7C). In contrast, three rats in the 7-day cohort had quantifiable RPV in iliac nodes (639, 5423 and 20,620 ng/g). Interestingly, there was no significant difference between the RPV concentrations in iliac nodes across the three different time points. In all cases, RPV concentrations in iliac, external lumbar and axillary nodes at the 28-day time point were below the LOQ of the HPLC system. At 56 days, only one animal had quantifiable RPV in the iliac node, while two animals had drug quantified in the external lumbar node and similarly two had drug quantified in the axillary nodes. Once again, in the IM study arm, there were no statistical differences in RPV concentrations in any of the lymph nodes across the different time points tested. There were also no statistical differences in RPV concentrations across common lymph nodes in the two study arms. In the only equivalent study carried out in rats, in which male rats received either 5 or 20 mg/kg RPV LA *via* IM, no drug was detectable in lymphoid tissues (thymus or spleen) of the rats 8 weeks post-dose [35]. No lymph nodes were extracted from the animals used in that study however. In a parallel study in beagle dogs, RPV was detected, ranging between 6 and 19 ng/mg in lymph nodes, 1 month post-IM dose of RPV LA [35]. The results presented here may be considered comparable to those reported previously but using a novel delivery vehicle. Importantly, as there were no significant differences in the levels of drug in the iliac nodes across various time points, this suggests the drug exposure is sustained, at least up to 8 weeks post-administration in those nodes.

In considering the possibility for self-application of MAPs, our research team and others have already published user feasibility studies, with results indicating that MAPs can be reproducibly applied to the skin without the need for complex applicators or specialist personnel [[41], [42], [43]]. In addition to this, recent work from our laboratory has deduced that larger, multi-array patches (16 cm^2^ MAPs) can be successfully self-administered by individuals, and to comparable depths as smaller patches with a single array, following appropriate instruction [38]. The outcomes of that study are of particular importance and relevance as it was the first study to investigate the self-application of “large” MAPs. Moving toward patient usage, comprehensive skin irritation and dermal toxicity evaluations must be carried out, followed by user feasibility studies which will enhance MAP concept and design and inform subsequent design approaches for these innovative drug delivery devices.

## 4 Conclusions

This work documents a proof of principle study outlining the formulation of novel dissolving MAPs for intradermal delivery of RPV. This is the first time that an ARV nanosuspension has been incorporated into a MAP delivery format and the results illustrate the potential delivery capabilities of such a novel device. RPV LA administration *via* MAP or IM led to comparable plasma, localised tissue and lymphoid tissue distribution results. Moreover, it has been cautiously estimated that an RPV LA MAP of approximately 28 cm^2^ could maintain therapeutic plasma levels of RPV over 7 days in humans. This work supports the potential future use of MAPs in the needle-free delivery of other long-acting ARV drugs, thus eradicating the necessity for daily oral medication regimens and eliminating the risks associated with needle-stick injuries. The outcomes of the current study have now been utilized to inform the target product profile for ARV MAPs. Formulation optimisation, stability evaluations, comprehensive preclinical and clinical pharmacokinetic studies, physiologically-based pharmacokinetic modelling and patient usability/acceptability studies are now necessary to fully realize the potential of these novel delivery platforms.
